# Depression, combined visual and hearing impairment (dual sensory impairment): a hidden multi-morbidity among the elderly in Residential Care in India

**DOI:** 10.1038/s41598-021-95576-5

**Published:** 2021-08-10

**Authors:** Srinivas Marmamula, Thirupathi Reddy Kumbham, Satya Brahmanandam Modepalli, Navya Rekha Barrenkala, Ratnakar Yellapragada, Rahul Shidhaye

**Affiliations:** 1 Allen Foster Community Eye Health Research Centre, Gullapalli Pratibha Rao International Centre for Advancement of Rural Eye care, L V Prasad Eye Institute, Hyderabad, 500034 India; 2 Brien Holden Institute of Optometry and Vision Science, L V Prasad Eye Institute, Hyderabad, 500034 India; 3 Wellcome Trust/Department of Biotechnology India Alliance, L V Prasad Eye Institute, Hyderabad, 500034 India; 4 School of Optometry and Vision Science, University of New South Wales, Sydney, Australia; 5 Pravara Institute of Medical Sciences, Loni, Maharashtra India

**Keywords:** Eye diseases, Psychiatric disorders, Health care, Medical research

## Abstract

To report the prevalence of depression and its association with combined visual (VI) and hearing impairment (HI) in the elderly in residential care in India. Participants aged ≥ 60 years were recruited from 41 homes. Data on personal and sociodemographic information were obtained. Visual acuity was measured using the logMAR chart. Patient Health Questionnaire (PHQ-9) was used to assess depression, and the Hearing Handicap Inventory for the Elderly Screening (HHIE) was administered to assess hearing status. Data of all 867 eligible elderly participants were analysed. The mean age of the participants was 74.2 years (standard deviation: 8.2 years) and included 537 (61.9%) women and 117 (13.5%) participants who had no education. The prevalence of depression was 60.0% (95% CI: 45.2–73.4) in the elderly with combined VI and HI compared to 20.9% (95% CI:14.4–28.8) among those with VI only and 37.8% (95% CI: 26.6–46.5) among those with HI only. On multiple logistic regression analyses, depression was approximately 5 times higher among the participants with DSI after adjusting for other covariates. Six out of ten elderly with combined HI and VI had depression highlighting the need for screening and referral when elderly present with combined vision and hearing loss.

## Introduction

The Global Burden of Disease estimated that 45.7 million individuals are affected by depressive disorders in India^1^. Depression is an emerging public health challenge affecting over a third of the elderly population in India^2,3,4,5,6^. A more recent population-based study done in northern India has reported even a higher prevalence of 41% of elderly with depression in a rural community^7,8^. The multi-country World Health Organization (WHO) Study on Global AGEing and Adult Health (SAGE)—wave 1, conducted during 2007–2010 reported a higher prevalence of 27.1% among those aged 50 years or older in India compared to 23.7% in Mexico, 15.6% in Russia, 11% in Ghana, 6.4% in South Africa and the least prevalence of 2.6% in China^9^.

Depression is known to be associated with vision impairment (VI) in the elderly^10^. Similarly, depression is also reported to be associated with hearing impairment (HI)^11,12^. Most studies on depression in the elderly report effects of either VI and depression or HI and depression independent of one another. This overlooks the possibility that VI and HI may, and often do, occur together in the elderly. This combination of VI and HI in an individual is called dual sensory impairment (DSI)^13^. A rapid assessment study of VI and HI conducted among 50 years or older population reported a DSI prevalence of 4.7%^14^. A community-based study from India identified that over one-third of the elderly had DSI^15^. Only one study in India revealed an association between the level of anxiety or depression and the level of physical or sensory functional difficulty which included HI and VI^16^.

India is witnessing a demographic shift with an increasing proportion of elderly in the population. Every fifth person would be elderly by the year 2050. The societal changes, increasing urbanization, and moving away from the traditional joint family system to nuclear families are resulting in the elderly people either living alone or only with a spouse or moving into homes for the aged. There is an increase in the number of homes in recent years. Understanding the health and wellbeing status among the elderly in residential care is essential to plan strategies for healthy aging in India.

Studies of the elderly in residential or home care reported a 13.4–86% prevalence of depression in India^17–19^. However, we are unaware of any studies of depression among those with combined VI and HI in the elderly living in residential care in India. Given the increase in the elderly population in India associated with changing living arrangements, research into the health status of the elderly in residential care assumes significance.

With this background, the Hyderabad Ocular Morbidity in Elderly Study (HOMES) sought to investigate the prevalence, causes, and risk factors for VI among elderly individuals living in residential care facilities in Hyderabad, India^20^. The secondary objective of this study was to assess the prevalence of depression and its association with VI, HI, and DSI. We had earlier reported that close to a third of the elderly had VI with over 80% VI being due to avoidable causes^21^. In this paper, we report the co-morbidity of depression with VI, HI, and combined VI and HI (DSI) among elderly living in residential homes in Hyderabad, India.

## Methods

### Ethics approval

The study protocol was approved by the institutional review board of the Hyderabad Eye Research Foundation, L V Prasad Eye Institute, Hyderabad, India. The study was carried out according to the tenets of the Declaration of Helsinki. The elderly residents were enrolled in the study after obtaining written informed consent.

### Study participants and assessments

A total of 1,515 participants from 41 homes for the aged in Hyderabad were recruited and 1,182 of them were examined in HOMES. The selection of homes and eligibility criteria used, and socio-demographic profile of the participants are reported in our previous publications^20,21^. In short, 46/76 (60.5%) homes for the aged in the Greater Hyderabad Municipal Corporation (GHMC) in the south Indian state of Telangana are included in the study (including 5 for the pilot study). The residents who were aged ≥ 60 years at the time of enumeration, residing in these homes for at least one month and agreed to participate were included in the study. As reported in our previous publications, homes are classified into three categories, (a) privates homes where the elderly or their kin pay a monthly or annual user fee, (b) Aided/Partially subsidized homes where the elderly or their kin pay a only a part of the user fee and rest of the amount is met by philanthropic support or other funding sources by the homes and (c) Free homes where the elderly need not pay any user fee as homes are supported by external funding sources. There are no prerequisites for entry into any of these homes expect that age criteria of 60 years or older but often people younger than 60 years are also admitted. The number of residents in homes vary from less than 30 to over 100 people in few homes.

The personal and demographic information of participants was collected, including their age, gender, highest education level, marital status, mobility status, and the number of years of stay in the home. The questionnaire also included information on self-report for diabetes and or hypertension. Trained field investigators administered a Hindi Mini Mental State Examination questionnaire which is a validated version for Indian population Mini-mental state examination (MMSE) assessment questionnaire^22–24^. For all those individuals whose HMSE scores were greater than or equal to 20, the Patient Health Questionnaire (PHQ-9) was used to assess depression, and the Hearing Handicap Inventory for the Elderly Screening (HHIE) were administered to assess hearing status^25,26^. The PHQ-9 questionnaire is validated for use in Indian population^27^. PHQ-9 and HHIE were not administered if the HMSE score was less than 20 (suggestive of mild cognitive impairment)^22^. After the interview, a detailed eye examination was done as reported in our earlier publications^20,21,28^. This assessment included visual acuity assessment for distance and near using a standard logMAR (logarithm of the minimum angle of resolution) chart under good illumination. All the interviews were audio-recorded as a part of quality control measure. Interviews and clinical assessments are always done on different days to ensure that the elderly participants get adequate rest between the assessments. Interviews were always done first followed by clinical assessments.

### Outcomes

In our study, a participant with a PHQ-9 score of 10 or higher was considered as having moderate depression, in line with published criteria for depression using the PHQ-9 questionnaire^26^. Visual impairment (VI) was defined as presenting visual acuity worse than 6/18 in the better eye^21^.Hearing impairment (HI) was defined as HHIE Score greater than ten as recommended by previous authors^25^. Dual Sensory Impairment (DSI) was considered to be present if a participant had both VI and HI.

### Other covariates

Independent variables included age group (60–69, 70–79, and ≥ 80 years), gender (male, female), education (higher education (Graduation and above), school education only (1–12 years of education), or no education), marital status (married, widowed/separated/single), mobility status based on self-report and interviewer’s observation (independently mobile, mobile with assistance), number of years of stay at the home (< 5 years, 5–9 years, ≥ 10 years), self-report on diabetes (Present/Absent) and hypertension (Present/Absent).

### Statistical analysis

Statistical analyses were conducted using Stata, version 14 (Stata Corp). The prevalence estimates were calculated and presented with 95% confidence intervals (CI). Pearson χ2 test was used for categorical variables. The dependent variable, depression was dichotomized: no depression = 0, depression = 1. In multiple logistic regression analysis, depression was used as an outcome variable and its association with DSI and other sociodemographic covariates (age, gender, level of education, marital status, years of stay at the home), systemic health factors (hypertension, diabetes, and mobility status) were assessed. All the variables were included in the model at a time. The selection of covariates in the model was based on previous studies that reported on Depression in India. In addition, the variables relevant to the elderly in residential care were included in the model. The Hosmer–Lemeshow goodness of fit test was used to assess the model fit. Variance inflation factors (VIF) were used to test for collinearity between the covariates after fitting a multiple regression model. The adjusted odds ratio (OR) with 95% confidence intervals (CI) was presented. For all analyses, the statistical significance was assessed at the conventional level of P less than 0.05 (two-tailed), however, exact P values were reported.

## Results

### Characteristics of the participants

**Figure 1:**
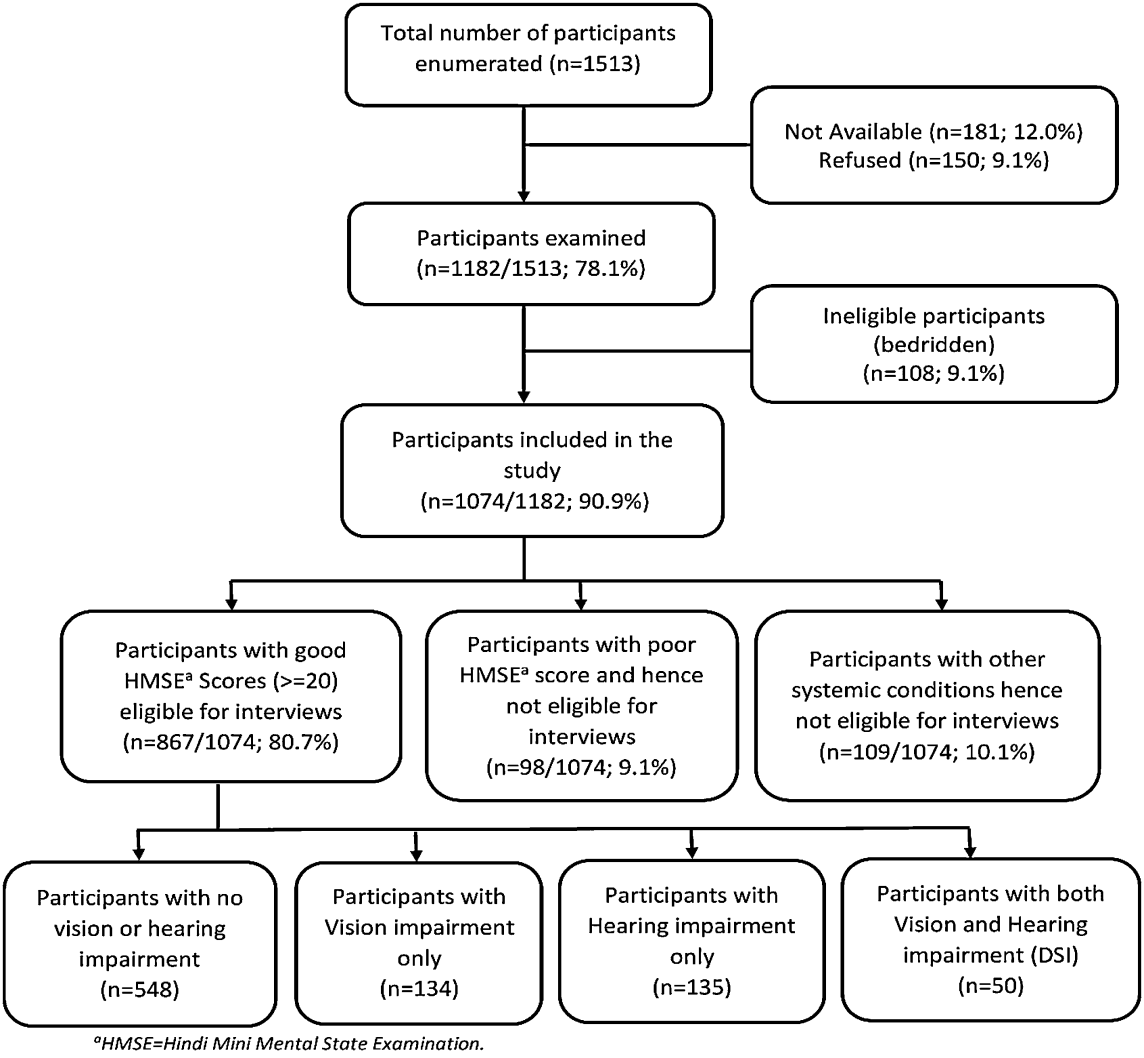
Flowchart showing the study participants with inclusion and exclusion criteria (n = 867).

The data of all 867 eligible elderly participants were included in the analyses (Fig. 1). The mean ± standard deviation (SD) age of the participants was 74.2 ± 8.2 years. There were 537 (61.9%) women, 117 (13.5%) participants who had no education, 559 (64.5%) participants with school education and 191 (22.0%) participants who had higher education. In terms of marital status, 669 (77.2%) individuals were either widowed, separated, or single. In all, 562 (64.8%) participants were living in a residential home for less than 5 years, whereas 140 (16.1%) were living in a home for ten years or more. Diabetes was reported by 263 (30.2%) participants while hypertension was reported by 518 (59.7%) participants. Difficulty in independent mobility was reported by 254 (29.3%) participants. In total, 681 (78.6%) participants were using spectacles and only 28 (3.2%) participants were using hearing aids. DSI was present in 50 participants (5.8%; 95% CI: 4.3–7.5). The socio-demographic characteristics of the study population are shown in Table 1.

**Table 1:** Illustrates the characteristics of the study sample stratified by presence or absence of a depression (n = 867) – Univariable analysis.

	Total in the group	Depression	
	n	n	Prevalence % (95% CI)
**Age group (years)**			
60–69	263	53	20.2 (15.5–25.5)
70–79	345	72	20.9 (16.7–25.5)
80 and above	259	70	27.0 (21.7–32.9)
**Gender**			
Male	330	77	23.3 (18.9–28.3)
Female	537	118	22.0 (18.5–25.7)
**Education**			
Higher education	192	30	15.6 (10.8–21.5)
School education	559	125	22.4 (19.0–26.0)
No education	116	40	34.5 (25.9–43.9)
**Marital status**			
Married	198	46	23.2 (17.5–29.7)
Widowed/Separated/Single	669	149	22.3 (19.2–25.6)
**Years of residence in home**			
< 5 years	562	138	24.6 (21.0–28.3)
5–9 years	165	31	18.8 (13.1–25.6)
> = 10 years	140	26	18.6 (12.5–26.0)
**Diabetes**			
No	604	140	23.2 (19.9–26.8)
Yes	263	55	20.9 (16.2–26.3)
**Hypertension**			
No	349	68	19.5 (15.5–24.0)
Yes	518	127	24.5 (20.9–28.5)
**Mobility status**			
Independent	613	120	19.6 (16.5–22.9)
Poor mobility	254	75	29.5 (24.0–35.6)
**Dual sensory impairment**			
No	817	165	20.2 (17.5–23.1)
Yes	50	30	60.0 (45.2–73.6)
	**867**	**195**	**22.5 (19.7–25.4)**

### Prevalence and risk factors for depression

**Figure 2:**
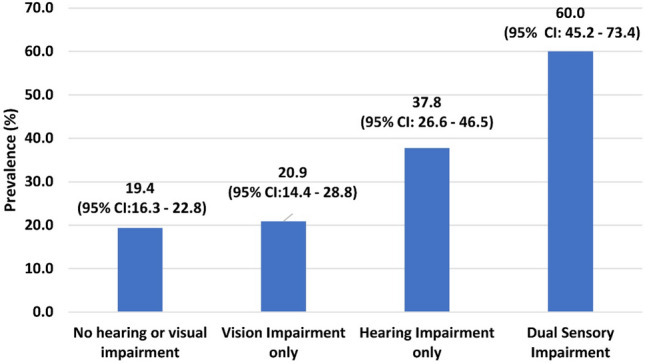
Prevalence of depression with levels of sensory loss and combined sensory loss.

The prevalence of depression among the elderly with DSI was 60.0% (95% CI: 45.2–73.4). It was 20.9% (95% CI: 14.4–28.8) among those with VI and 37.8% (95% CI: 26.6–46.5) among those with HI. The prevalence of depression was the least among those with no sensory impairment (19.4%; 95% CI: 16.3–22.8). (Fig. 2) In the overall sample, 195 participants had depression (22.5%; 95% CI: 19.8–25.4). On univariable analyses, the prevalence of depression was significantly higher among those without education (p < 0.01), poor mobility (p < 0.01), HI (p < 0.01), VI (p < 0.01), and DSI (p < 0.01). Age, gender, marital status, number of years of living in a home, diabetes, or hypertension were not associated with depression. (Table 1).

In multiple logistic regression analyses, depression was approximately five times higher among the participants with DSI (OR: 4.90; 95% CI: 2.63–9.14) after adjusting for other covariates. The odds of depression were also higher among those with no education (OR: 1.39; 95% CI: 1.46–4.60) compared to participants with higher education while it was of borderline significance (OR: 1.60; 95% Cl: 1.00–2.56; p = 0.05) among those with school education. Participants with poor mobility were more likely to have depression (OR: 1.54; 95% CI: 1.06–2.23) (Table 2).

**Table 2:** Multiple logistic regression assessing the factors associated with depression (n = 867) among the elderly in residential care in India.

	Adjusted odds ratio (95% CI)	P values
**Age group (years)**		
60–69	Reference	
70–79	0.95 (0.62–1.44)	0.798
80 and above	1.07 (0.67–1.70)	0.773
**Gender**		
Male	Reference	
Female	0.90 (0.60–1.32)	0.599
**Education**		
Higher education	Reference	
School education	1.60 (1.00–2.56)	0.05
No education	1.39 (1.46–4.60)	< 0.01
**Years of residence**		
< 5 years	Reference	
5–9 years	0.72 (0.45–1.14)	0.156
> = 10 years	0.72 (0.44–1.18)	0.199
**Marital status**		
Married	Reference	
Widowed/separated/single	0.88 (0.58–1.32)	0.53
**Diabetes**		
No	Reference	
Yes	0.94 (0.65–1.38)	0.75
**Hypertension**		
No	Reference	
Yes	1.36 (0.95–1.96)	0.09
**Mobility status**		
Independent	Reference	
Poor mobility	1.54 (1.06–2.23)	0.022
**Dual sensory impairment**		
No	Reference	
Yes	4.9 (2.63–9.14)	< 0.01

## Discussion

One out of every four elderly in residential care in Hyderabad, India had depression. The elderly with DSI were five times more at risk for depression. Also, depression was higher among those with single sensory impairment than in those without any sensory impairment. Several factors may have contributed to their depression^4,9,29,30^. Apart from a singular or double sensory impairment, other health issues or psychosocial and emotional factors are reported to be associated with depression^4^. We did not find any association between hypertension or diabetes and depression. Poor mobility was also associated with depression after controlling for other covariates. A recent study among the homes for the aged in Mangalore city in South India reported that nearly half of the residents had depression^31^. Similar to our study, there was no association between depression and gender. The association between depression and gender are inconsistently reported in the community-based studies in India. A systematic review that included elderly in the communities and the LASI (Longitudinal Ageing Study in India (LASI) Wave 1) that also included the elderly in the communities did not report the any association between depression and gender^32,33^. However, few studies done in a community setting in the northern in India and a systematic review reported a higher odds for depression among women^3,7,34^.

As vision and hearing are vital senses that one heavily depends on and is habituated to using routinely, their lack may be perceived as a grievous loss. Thus, one’s inability to see clearly and or hear properly may predispose the elderly to depression. While DSI can lead to loss of independence, difficulties with self-care can adversely affect one’s emotional well-being^35–38^. As the elderly in residential care are relatively more dependent on others for their daily living as compared to persons living at home with their family, it is likely that the effect of DSI can compound their difficulties and worsen the level of depression compared to those who live with their family in the community setting. No comparative study has reported this in India.

In developed countries, DSI is reported to be a significant challenge in the elderly^39^. The prevalence of DSI ranged from 9.7 to 33.9% among the elderly in residential care in a study of four countries—Canada, the United States, Finland, and Belgium^40^. Over 14% of the elderly were reported to have DSI in the United States^41^. Also, the impact of DSI on depression and quality of life has been reported in developed countries^35–38,42–46^. Though we found a lower prevalence of DSI (5.8%) in our study, 60% of those with DSI had depression. However, due to differences in the study setting, methods used and time of the study, direct comparison across the studies may not be appropriate.

Also, studies done in the communities cannot be extrapolated to the elderly in residential care. The elderly in residential care have a limited scope of activities given that essential activities such as cooking, and cleaning are attended to by employees or caretakers in the home. These individuals are staying away from one’s family due to social or family reasons and this may likely lead to loneliness, isolation, anger, anxiety, feelings of loss, depression, and so on. Also, mobility is restricted to the confines of the home, with the elderly seldom stepping out on their own to visit any place or for shopping. The situation worsens if they develop a non-communicable health condition compounded by VI or HI, often both worsening their quality of life.

Like any other psychosocial entity, depression is a complex phenomenon interacting with several other factors. We must recognize the factors leading to depression that are avoidable at the primary level before resorting to medical or other intervention for the depression. In our earlier publication based on the same population, we had reported that over 80% of the vision loss is due to avoidable causes^21^. This can be dealt with on a priority basis by the provision of spectacles and cataract surgery. These interventions may result in decreasing the burden of depression. Though a large proportion of the elderly was using spectacles for their vision loss, very few people reported using a hearing aid^47^. This implies that a higher priority given to eye health, with easy availability and relative ease of access to the services. As reported earlier, we had found that a large proportion of those using spectacles continues to have poor vision indicating a need for a regular eye examination and replacement of the spectacles^47^.

Oswley and colleagues reported improvement in the quality of life and decline in the level of depression as a result of providing spectacles to nursing home residents in the United States^48^. However similar studies are not reported from India. Hearing loss leads to difficulty in communication and may result in depression^12,49–53^. A recent systematic review indicated a significant improvement in the quality of life after wearing a hearing aid^54^. A cohort that included over 110,000 elderly participants concluded that wearing a hearing aid delayed the onset of dementia and or anxiety^55^. Several non-pharmacological interventions for depression of the elderly have been reported in India^4^. These include yoga, meditation, physical exercise, and psychological therapies^56–58^.To address depression, a few innovative programmes have been tested and found successful in India^59–61^.

In the current scenario, assessments and services are provided by different specialists. We need to adopt a holistic approach to jointly address the multi-morbidity associated with DSI and depression. This is quite unlike adopting the unilateral method of care when treating a single sensory impairment such as only VI or only HI. In India, these two conditions are independently managed by different domain experts. Thus, the patient is treated in isolation, with minimum scope for a further referral at the time of consultation. The absence of multi-specialty clinic-based referral calls for a more integrated multi-morbidity focused approach to understand elderly health needs and to provide comprehensive services.

Studies have also shown that social isolation, lack of family care and support are associated with depression in the elderly. India is moving away from a joint family towards a nuclear family^62,63^. Such social change brings on depression, necessitating efforts to address this issue through newer methods and counselling.

We noted a difference in the prevalence of depression across the studies which may be attributed in part to the methodological difference adopted. The Geriatric Depression Score and PHQ 9 are the most commonly used methods for assessing depression. Few studies on DSI restricted to ‘self-report’ on vision impairment instead of objective assessment of visual acuity. Similarly, a few studies have used self-report of hearing loss while other studies used objective audiometric assessments.

Large sample size from 41 homes with a good response rate is a strength of our study. All assessments were done in the comfort of the participants at a slow pace to facilitate greater participation and better responses. All the interviews were audio-recorded with consent which helped us to maintain the quality of the interview. Our results can be extrapolated to the homes located in urban areas in India and can be used for planning purposes. We used HMSE as a screening tool with a cutoff of 20 to recruit participants for assessments of DSI. While this might have given more reliable and consistent responses, it may have underestimated the prevalence of depression and other psychiatric disorders in our population. In our study, the diagnosis of VI was based on a standard objective measurement of visual acuity but HI was diagnosed based on a self-report on responses to the questionnaire and not based on audiometry. Similarly, assessment of depression was also based on PHQ-9, a screening tool and not diagnostic assessment. We also excluded the participants who were bedridden which could have underestimated the prevalence of depression in our study population.

In conclusion, depression and DSI are common in the elderly living in residential care. Since patients with DSI are at higher risk of developing depression, assessment of depression should be performed as part of the routine practice in eye and ear examination clinics in India. A multi-pronged approach to address this important multi-morbidity is critical to improve the quality of life and facilitate healthy aging for elderly in India.

## References

[1] India State-Level Disease Burden Initiative Mental Disorders, C. The burden of mental disorders across the states of India: the Global Burden of Disease Study 1990–2017. *Lancet Psychiatry* **7**, 148–161. 10.1016/S2215-0366(19)30475-4 (2020). 10.1016/S2215-0366(19)30475-4 PMC7029418 31879245

[2] Pilania M, Bairwa M, Kumar N, Khanna P, Kurana H (2013). Elderly depression in India: An emerging public health challenge. Australas. Med. J..

[3] Pilania M (2019). Prevalence of depression among the elderly (60 years and above) population in India, 1997–2016: a systematic review and meta-analysis. BMC Public Health.

[4] Grover S, Malhotra N (2015). Depression in elderly: A review of Indian research. J. Geriatr. Ment. Health.

[5] Shidhaye R, Gangale S, Patel V (2016). Prevalence and treatment coverage for depression: a population-based survey in Vidarbha, India.. Soc. Psychiatry Psychiatr. Epidemiol..

[6] Devi ES (2007). Elderly and depression. Nurs. J. India.

[7] Sahni B, Bala K, Kumar T, Narangyal A (2020). Prevalence and determinants of geriatric depression in North India: A cross-sectional study. J. Fam. Med. Primary Care.

[8] Sinha SP, Shrivastava SR, Ramasamy J (2013). Depression in an older adult rural population in India. MEDICC Rev..

[9] Anand A (2015). Understanding depression among older adults in six low-middle income countries using WHO-SAGE survey. Behavioral Health.

[10] Tetteh J (2020). Visual impairment and social isolation, depression and life satisfaction among older adults in Ghana: analysis of the WHO's Study on global AGEing and adult health (SAGE) Wave 2. BMJ Open Ophthalmol..

[11] Niazi Y, Ejaz B, Muazzam A (2020). Impact of hearing impairment on psychological distress and subjective well-being in older adults. Pak. J. Med. Sci..

[12] Cosh S, Helmer C, Delcourt C, Robins TG, Tully PJ (2019). Depression in elderly patients with hearing loss: current perspectives. Clin. Interv. Aging.

[13] Wittich W, Southall K, Sikora L, Watanabe DH, Gagné J-P (2013). What’s in a name: Dual sensory impairment or deafblindness?. Br. J. Vis. Impair..

[14] Bright T (2020). Rationale and feasibility of a combined rapid assessment of avoidable blindness and hearing loss protocol. PLoS ONE.

[15] R D, Kasthuri A (2012). Visual and hearing impairment among rural elderly of south India: a community-based study. Geriatr. Gerontol. Int..

[16] Wallace S, Mactaggart I, Banks LM, Polack S, Kuper H (2020). Association of anxiety and depression with physical and sensory functional difficulties in adults in five population-based surveys in low and middle-income countries. PLoS ONE.

[17] Singh AP, Kumar KL, Reddy CM (2012). Psychiatric morbidity in geriatric population in old age homes and community: A comparative study. Indian J. Psychol. Med..

[18] Tiwari K, Aggarwal P, Kakkar R, Tiwari A (2020). Moving out of shadows: Depression among the elderly in dehradun district of Uttarakhand, India.. J. Lifestyle Med..

[19] Kumar R, Satapathy S, Adhish V, Nripsuta S (2017). Study of psychiatric morbidity among residents of government old age homes in Delhi. J. Geriatr. Ment. Health.

[20] Marmamula S (2020). Hyderabad ocular morbidity in elderly study (HOMES)—Rationale, study design and methodology.. Ophthalm. Epidemiol..

[21] Marmamula S (2021). Prevalence and risk factors for visual impairment among elderly residents in 'homes for the aged' in India: The Hyderabad Ocular Morbidity in Elderly Study (HOMES). Br. J. Ophthalmol..

[22] Folstein MF, Folstein SE, McHugh PR (1975). Mini-mental state: A practical method for grading the cognitive state of patients for the clinician. J. Psychiatr. Res..

[23] Ganguli M (1995). A Hindi version of the MMSE: the development of a cognitive screening instrument for a largely illiterate rural elderly population in India. Int. J. Geriatr. Psychiatry.

[24] Pandav R, Fillenbaum G, Ratcliff G, Dodge H, Ganguli M (2002). Sensitivity and specificity of cognitive and functional screening instruments for dementia: The Indo-US Dementia Epidemiology Study. J. Am. Geriatr. Soc..

[25] Ventry IM, Weinstein BE (1982). The hearing handicap inventory for the elderly: a new tool. Ear Hear.

[26] Kroenke K, Spitzer RL, Williams JB (2001). The PHQ-9: validity of a brief depression severity measure. J. Gen. Intern. Med..

[27] Kochhar P, Rajadhyaksha S, Suvarna V (2007). Translation and validation of brief patient health questionnaire against DSM IV as a tool to diagnose major depressive disorder in Indian patients. J. Postgrad. Med..

[28] Marmamula S (2020). Falls and visual impairment among elderly residents in 'homes for the aged' in India. Sci. Rep..

[29] A, R. A. & Noronha, J. A. Depression among older adults: a systematic review of South Asian countries. doi:10.1111/psyg.12644 (2020). 10.1111/psyg.12644 33319427

[30] Arokiasamy P, Verma U, Kowal P (2014). On depression in an older adult population of rural India. MEDICC Rev..

[31] Kumar S, Joseph S, Abraham A (2021). Prevalence of depression amongst the Elderly population in old age homes of Mangalore city. J. Fam. Med. Primary Care.

[32] Barua A, Ghosh MK, Kar N, Basilio MA (2011). Prevalence of depressive disorders in the elderly. Ann. Saudi Med..

[33] Muhammad T, Meher T (2021). Association of late-life depression with cognitive impairment: evidence from a cross-sectional study among older adults in India. BMC Geriatr..

[34] Pilania M, Bairwa M, Khurana H, Kumar N (2017). Prevalence and Predictors of Depression in Community-Dwelling Elderly in Rural Haryana, India. Indian J. Commun. Med..

[35] Bouscaren N, Yildiz H, Dartois L, Vercambre MN, Boutron-Ruault MC (2019). Decline in instrumental activities of daily living over 4-Year: The association with hearing, visual and dual sensory impairments among non-institutionalized women. J. Nutr. Health Aging.

[36] Brennan M, Horowitz A, Su YP (2005). Dual sensory loss and its impact on everyday competence. Gerontologist.

[37] Kiely KM, Anstey KJ, Luszcz MA (2013). Dual sensory loss and depressive symptoms: The importance of hearing, daily functioning, and activity engagement. Front. Hum. Neurosci..

[38] McDonnall MC (2009). The effects of developing a dual sensory loss on depression in older adults: A longitudinal study. J. Aging Health.

[39] Berry P, Mascia J, Steinman BA (2004). Vision and hearing loss in older adults: "Double trouble". Care Manag. J..

[40] Guthrie DM, Declercq A, Finne-Soveri H, Fries BE, Hirdes JP (2016). The health and well-being of older adults with dual sensory impairment (DSI) in four countries. PLoS ONE.

[41] Swenor BK, Ramulu PY, Willis JR, Friedman D, Lin FR (2013). The prevalence of concurrent hearing and vision impairment in the United States. JAMA Intern. Med..

[42] Chou KL (2008). Combined effect of vision and hearing impairment on depression in older adults: Evidence from the English Longitudinal Study of Ageing. J. Affect. Disord..

[43] Chia EM (2006). Association between vision and hearing impairments and their combined effects on quality of life. Arch. Ophthalmol..

[44] Lupsakko T, Mäntyjärvi M, Kautiainen H, Sulkava R (2002). Combined hearing and visual impairment and depression in a population aged 75 years and older. Int. J. Geriatr. Psychiatry.

[45] Capella-McDonnall ME (2005). The effects of single and dual sensory loss on symptoms of depression in the elderly. Int. J. Geriatr. Psychiatry.

[46] Schneider JM (2011). Dual sensory impairment in older age. J. Aging Health.

[47] Marmamula S (2020). Uncorrected refractive errors for distance among the residents in 'homes for the aged' in South India-The Hyderabad Ocular Morbidity in Elderly Study (HOMES). Ophthalm. Physiol. Opt..

[48] Owsley C (2007). Effect of refractive error correction on health-related quality of life and depression in older nursing home residents. Arch. Ophthalmol..

[49] Boi R (2012). Hearing loss and depressive symptoms in elderly patients. Geriatr. Gerontol. Int..

[50] Choi JS (2016). Association of Using Hearing Aids or Cochlear Implants With Changes in Depressive Symptoms in Older Adults. JAMA Otolaryngol Head Neck Surgery.

[51] Dalton DS (2003). The impact of hearing loss on quality of life in older adults. Gerontologist.

[52] Gopinath B (2009). Depressive symptoms in older adults with hearing impairments: the Blue Mountains Study. J. Am. Geriatr. Soc..

[53] Keller BK, Morton JL, Thomas VS, Potter JF (1999). The effect of visual and hearing impairments on functional status. J. Am. Geriatr. Soc..

[54] Ferguson MA (2017). Hearing aids for mild to moderate hearing loss in adults. Cochr. Database Syst. Rev..

[55] Mahmoudi E (2019). Can Hearing Aids Delay Time to Diagnosis of Dementia, Depression, or Falls in Older Adults?. J. Am. Geriatr. Soc..

[56] Raj D, Santhi S, Sapharina GJS (2020). Effectiveness of neurobic exercise program on memory and depression among elderly residing at old age home. J. Complem. Integrat. Med..

[57] Chobe S, Chobe M, Metri K, Patra SK, Nagaratna R (2020). Impact of Yoga on cognition and mental health among elderly: A systematic review. Complement. Ther. Med..

[58] Cramer H, Lauche R, Langhorst J, Dobos G (2013). Yoga for depression: a systematic review and meta-analysis. Depress Anxiety.

[59] Patel V (2017). The Healthy Activity Program (HAP), a lay counsellor-delivered brief psychological treatment for severe depression, in primary care in India: a randomised controlled trial. Lancet.

[60] Shidhaye R (2017). The effect of VISHRAM, a grass-roots community-based mental health programme, on the treatment gap for depression in rural communities in India: a population-based study. Lancet Psychiatry.

[61] Maulik PK, Devarapalli S (2020). The Systematic Medical Appraisal Referral and Treatment Mental Health Project: Quasi-Experimental Study to Evaluate a Technology-Enabled Mental Health Services Delivery Model Implemented in Rural India..

[62] Mane AB (2016). Ageing in India: some social challenges to elderly care. J. Gerontol. Geriatr. Res..

[63] Yeolekar ME (2005). Elderly in India–needs and issues. J. Assoc. Physicians India.

